# Is There an Environmental Kuznets Curve for Food-Related Carbon Emissions? Evidence from China

**DOI:** 10.3390/foods15071251

**Published:** 2026-04-06

**Authors:** Zilong Xu, Jiehong Zhou, Kai Li

**Affiliations:** 1 China Academy for Rural Development, School of Public Affairs, Zhejiang University, 866 Yuhangtang Road, Hangzhou 310058, China; leoxzl@zju.edu.cn (Z.X.); runzhou@zju.edu.cn (J.Z.); 2 School of Economics, Qufu Normal University, Room 720, 80 Yantai North Road, Rizhao 276826, China

**Keywords:** food consumption, carbon footprint, threshold effects, Environmental Kuznets Curve, China

## Abstract

Background/Objectives: Rising incomes worldwide are reshaping dietary patterns and intensifying concern about the carbon impacts of household food consumption. This study examines how economic development influences food-related carbon emissions in China, with a focus on household food consumption behavior and dietary change. Methods: Using longitudinal household data from the China Health and Nutrition Survey covering 2004–2011, we apply a panel threshold regression model to identify nonlinear income effects on food-related carbon emissions. Results: Within the 2004–2011 sample, household income is positively associated with food-related emissions, but the marginal effect declines once income exceeds the estimated threshold. The baseline model identifies a single threshold at 6.5479 (95% confidence interval: [6.4965, 6.5917]), corresponding to 65,479 yuan in annual household income. The single-threshold test is significant at the 5% level (*p* = 0.035), and the adjusted R^2^ is 0.243. Income growth significantly increases the consumption of greenhouse-gas-intensive foods and associated emissions among low-income households, whereas food consumption patterns among high-income households are comparatively more stable within the sample period. Conclusions: These findings indicate that rising income can intensify food-related carbon pressure during China’s dietary transition, particularly through dietary upgrading among low-income households, but they do not provide direct evidence that household food emissions will stabilize automatically over time.

## 1. Introduction

Climate change caused by the massive emissions of greenhouse gases (GHG) has become an important environmental issue of global concern and research. Promoting carbon peaking and neutrality is an urgent task for all countries. The amount of GHG emissions from the food system is significant. According to IPCC’s research, the agriculture, forestry, and land use sectors contribute between 13% and 21% of GHG emissions [1]. China is the world’s second-largest energy consumer after the United States, with per capita consumption of pork, beef, poultry, and milk increasing threefold, tenfold, elevenfold, and twentyfold, respectively, from 1980 to 2009 [2]. Meanwhile, GHG emissions grew from 489 Mt carbon dioxide equivalents (CO_2_eq) in 1996 to 732 Mt CO_2_eq in 2010 [3]. He et al. [4] further pointed out that the food consumption structure of Chinese residents varies widely, and the food emissions and nutrition intake of different income groups show various characteristics. Therefore, clarifying the relationship between economic development and food emissions, and targeting interventions according to the characteristics and nutritional needs of households, are of great significance to solving the “food security, human health, and climate change” trilemma faced by developing countries represented by China. This understanding also has a far-reaching impact on the realization of the Sustainable Development Goals (SDG (https://www.un.org/sustainabledevelopment/sustainable-consumption-production/, accessed on 25 June 2025)) proposed by the United Nations.

Recent food and nutrition research suggests that rising income reshapes food demand through dietary upgrading rather than through a simple environmental curve. Studies in China and other settings show that income growth is associated with more diversified diets, rising animal-source food consumption, and changes in both nutritional quality and environmental pressure [4,5,6]. In the food literature, this process is increasingly framed as a joint nutrition-environment problem rather than as a purely environmental one [7,8,9,10]. Household-level heterogeneity, therefore, matters because the same increase in income can lead to different food choices, dietary quality outcomes, and emission consequences across regions and demographic groups [8].

At the same time, the food-related emissions literature does not point to a settled Environmental Kuznets Curve (EKC) pattern. Existing studies document rising carbon pressure from dietary upgrading and animal-source food expansion [10,11,12,13], while recent household-level evidence shows that income can also shape food-related carbon outcomes through specific behavioral channels such as food waste [14]. In this context, EKC should be treated as a hypothesis about nonlinear income-emissions relationships rather than as the organizing framework of the food literature. Classic EKC studies remain useful for motivating threshold analysis [15], but evidence from Cole and McCoskey [16] and Tonsor and Lusk [17] is drawn from mature meat markets and cannot be transferred mechanically to Chinese households during an active stage of dietary transition. Consistent with Bennett-type dietary upgrading, empirical studies for China show that income growth is associated with lower cereal dependence and stronger demand for animal-source foods and other quality-differentiated foods [5,6].

Taken together, three gaps remain. First, the food and food-consumption literature has documented dietary upgrading, but it rarely connects food consumption amount, food structure, dietary quality, and food-related emissions within one unified household framework. Second, many food-emissions studies rely on macro accounting, regional comparisons, or descriptive trends [11,18,19,20,21,22], leaving limited micro evidence on whether income effects differ across households at different stages of dietary transition. Third, the EKC literature asks whether emissions eventually decouple from growth, but it seldom links that nonlinear question to food-specific mechanisms such as animal-source-food expansion, diet quality, and household heterogeneity. A household-panel approach is therefore needed to analyze the food–environment relationship as a question of dietary transition rather than as a purely macro environmental curve.

Using CHNS household survey data from 2004 to 2011, we examine how income is associated with food-related carbon emissions during a critical stage of dietary transition in China. Our contribution is threefold. First, we provide household-level evidence on how income is associated with food consumption amount, dietary structure, and dietary quality after controlling for household characteristics. Second, we show that the income-emissions relationship is nonlinear, but the evidence supports threshold heterogeneity rather than a short-run EKC turning point. Third, by focusing on 2004–2011 as the active phase of dietary upgrading, we connect household food demand to the broader discussion of food-system sustainability. This Chinese evidence may also offer limited comparative insight for economies undergoing similar dietary transitions, but it should not be read as a general claim about developing countries.

The remainder of the paper is organized as follows. Section 2 describes the data, carbon-accounting approach, and empirical strategy. Section 3 presents the empirical results. Section 4 integrates the discussion and limitations of the study, with Section 4.1 interpreting the findings and Section 4.2 consolidating the main limitations. Section 5 presents the conclusion and policy implications, with Section 5.1 summarizing the core findings and Section 5.2 outlining the policy implications.

## 2. Data and Methodology

### 2.1. Data

#### 2.1.1. Accounting Methodology for Food-Related Carbon Emissions

Household carbon emissions are the CO_2_ emissions resulting from the whole life cycle of products and services for the household, including those associated with their manufacturing and eventual breakdown [23]. According to the boundary and scope of the calculation, it is usually categorized into direct carbon emissions and indirect carbon emissions [21]. Direct carbon emissions are generated by direct energy consumption, including household cooking, indoor cooling and heating, household appliances, private transportation, etc., and are generally measured by the emission-factor approach. Indirect carbon emissions consist of carbon emissions from other material and service needs in daily life, such as clothing, food, housing, transportation, etc., and are generally measured by the input–output approach or the life cycle approach.

Food-related carbon emissions are mainly indirect carbon emissions from production and transportation, and direct food-related carbon emissions behaviors, i.e., direct carbon emissions from storing and cooking at home, account for a relatively low percentage, and the data is difficult to obtain, so we focus on indirect food-related carbon emissions.

In indirect food-related carbon emissions, the input–output method is a bottom-up calculation method, which calculates the relationship between energy inputs and final products through a balancing equation, and is suitable for calculating greenhouse gas emissions at the macro scale, but cannot reflect the micro differences in household food consumption. The life cycle assessment (LCA) method measures the carbon emissions generated by different regions in different stages of production, processing, and distribution of food products, and the calculation process is more detailed and accurate, which is more suitable for micro-level carbon emissions research, but due to the different standards for defining the scope of the carbon emissions system, the results often lack uniformity among the research results. In this paper, we use the food emissions data summarized by Poore and Nemecek [24], which is mainly based on the LCA method (IPCC 2007), which adopts a wide range of literature, and the results are representative and robust, and can better measure food emissions in Chinese households.

#### 2.1.2. Data Source

**Figure A1:**
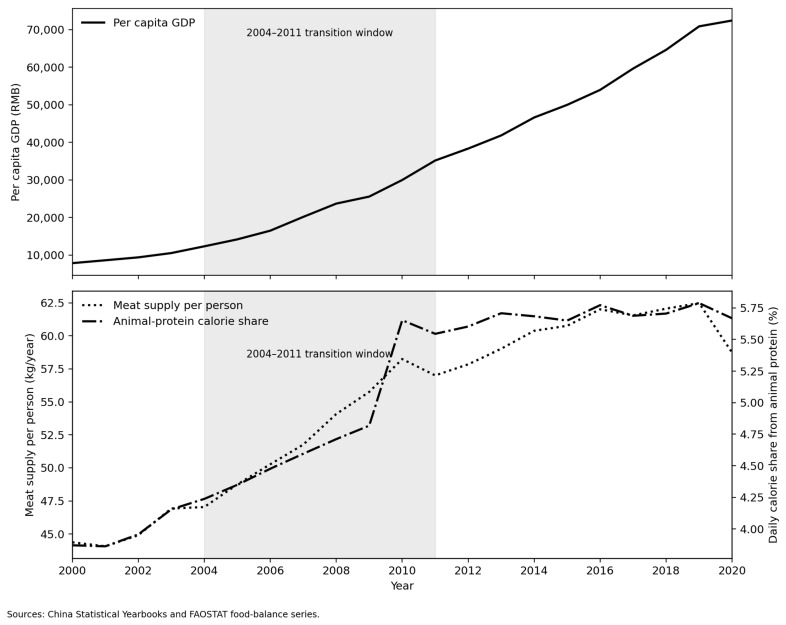
GDP per capita and food consumption transition in China.

The household panel data used in this study come from the China Health and Nutrition Survey (CHNS), which covers 12 provinces in eastern, central, and western China. CHNS is the only large-scale micro food-consumption survey in China and remains the most authoritative source for household-level food-consumption analysis. Food consumption is recorded using the 72 h dietary recall survey, and the reported intake is converted into annual household consumption on a comparable basis. We focus on the 2004, 2006, 2009, and 2011 waves because these four surveys provide a comparable micro sequence for constructing household food-related carbon emissions. This sample choice is also substantively meaningful. Public evidence compiled from China Statistical Yearbooks and FAOSTAT shows that 2004–2011 was the active phase of China’s dietary transition: GDP per capita rose from 12336 yuan to 35181 yuan, per capita meat supply increased from 47.0 kg to 57.0 kg, and the share of daily calories from animal protein rose from 4.24% to 5.54%. These descriptive trends are summarized in Figure A1 in Appendix A, which plots GDP per capita together with key indicators of China’sfood consumption transition. These shifts indicate a transition from basic calorie security toward more diversified and animal-source, food-intensive diets. Based on the data provided by Poore and Nemecek [24], household food-related carbon emissions are calculated by matching median CO_2_-equivalent values to the food-consumption records. Because these data do not include emissions from household food storage and cooking, the emissions analyzed here should be interpreted as indirect food-related carbon emissions. We fully acknowledge that the sample ends in 2011, which is an important limitation. At the same time, the 2015 CHNS does not provide food-consumption survey data for extending the present analysis, so the study cannot be naturally prolonged beyond 2011 within the same household-level micro framework.

In the panel data we used, due to the large range of fluctuations in total annual household income (RMB) as well as food emissions (tons), after shaving the samples with total annual household income less than 0, the total annual household income and food emissions are subjected to an upper and lower 1% shrinking tail treatment, resulting in a total of 8288 balanced panel samples. Referring to previous related studies, this paper uses household demographic characteristics as control variables, including household size (hhsize), the percentage of the number of children younger than six years old (age06), the percentage of the number of elderly people older than 65 years old (age65), the age of the head of the household (age), the square term of the age of the head of the household (age_sq), the number of years of education of the head of the household (edu), and the marital status of the head of the household (marriage), where being married is recorded as 1 and other status is recorded as 0.

#### 2.1.3. Trends in Food-Related Carbon Emissions in China

**Figure 1:**
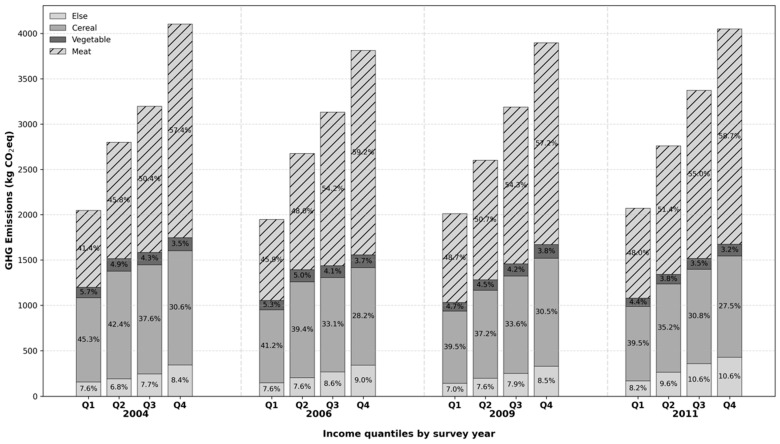
Trends in food-related carbon emissions from Chinese households.

Based on the panel data above, the trend of Chinese households’ food-related carbon emissions can be plotted as shown in Figure 1. It can be found that the total average food emissions (as shown by the black line) decreased a little bit from 2004 to 2006, and increased year by year from 2006 to 2011. By dividing the sample households into four groups based on the 25%, 50%, 75%, and 100% quantiles of total annual income, it can be found that overall food emissions from households with higher incomes are more than those of lower incomes. Furthermore, higher-income households account for a greater proportion of emissions from livestock meat (pork, beef, lamb, etc.) consumption, while lower-income households account for a greater proportion of emissions from cereal consumption. It is noteworthy that the share of carbon emissions from cereal consumption in all households has been decreasing year by year, while the share of livestock meat in households with incomes in the bottom 50% has been increasing, and the share of livestock meat in households with incomes in the top 50% has remained stable in general. The share in households with incomes in the top 25% has even shown a slight downward trend. The above trends show that food emissions vary greatly among households, and the composition of carbon emissions shows dynamic changes, which require further empirical analysis.

### 2.2. Threshold Regression Model

To study the food-related carbon emissions from households with different incomes, it is necessary to study the marginal effect of income on food emissions at different levels. The threshold model proposed by Hansen [25,26] enables “endogenous grouping” instead of “exogenous grouping”. The endogenous grouping can tell if there is a threshold effect, whereas the exogenous grouping can only give the points of income. However, because the threshold value under the null hypothesis is not identified, it is impossible to identify the asymptotic distribution of the threshold value estimator. The distribution of the LR statistics upon moments of the sample and its critical values cannot be tabulated. Hansen [26] suggests that the bootstrap method can be used to estimate the asymptotic *p*-value of the LR statistic, while related resampling inference procedures are discussed in [27]. The *p*-value of the threshold effect and its confidence interval can be used to determine whether the threshold effect exists and whether the threshold value obtained from the regression is reasonable to assume that the threshold value obtained from the regression is true [26].

(1) $${GHG}_{it}=\beta_{0}+\beta_{1}hhinc_{it}\cdot I\left(hhinc\leq \gamma \right)+\beta_{2}hhinc_{it}\cdot I\left(hhincpc>\gamma \right)+Z_{it}\theta +\mu_{i}+\lambda_{t}+\epsilon_{it}\;$$

where ${GHG}_{it}$ is the total food-related carbon emissions from household *i* in year t (tons); $hhinc_{it}$ is the total annual income of household *i* in year t (adjusted for the 2015 cpi); and γ is the threshold variable to be estimated. I(hhinc≤γ) is an indicator function denoting the income less than the threshold; I(hhinc>γ) is an indicator function denoting the income higher than the threshold; $Z_{it}$ is the control variable mentioned above; $\mu_{i}$ captures the household fixed effects that remain constant through the year; $\lambda_{t}$ captures the year’s fixed effect that is similar to all households; and $\epsilon_{it}$ is a random error term. To avoid the impact of food consumption correlations due to factors such as geographical tastes on the estimation results, we use cluster-robust standard error at the provincial level.

After estimating the threshold variable γ, it is important to determine whether there is a threshold and whether the estimated threshold is equal to the actual value. For the first question, the hypothesis of no threshold effect can be represented by the linear constraint $H_{0}:\beta_{1}=\beta_{2}$. The likelihood ratio test of $H_{0}$ is based on:

(2) $$F_{1}\left(\gamma \right)=\frac{S_{1}\left(\gamma \right)-S_{1}\left(\hat{\gamma }\right)}{{\hat{\sigma }}^{2}}$$

The asymptotic distribution of $F_{1}$ is non-standard and strictly dominates the $\chi_{k}^{2}$ distribution, which depends in general upon moments of the sample, and its critical values cannot be tabulated. Hansen [26] suggests that the bootstrap method can be used to estimate the asymptotic *p*-value to determine the threshold number of sample data.

If there is a threshold effect in the regression, we can further test whether the estimated threshold is equal to the actual value with the hypothesis $H_{0}:\;\gamma =\gamma_{0}$, the corresponding likelihood ratio statistics are as follows:

(3) $$LR\left(\gamma \right)=\frac{S_{1}\left(\gamma \right)-S_{1}\left(\hat{\gamma }\right)}{{\hat{\sigma }}^{2}}$$

At a significance level of α, when $LR\left(\gamma \right)\leq -2\ln\left(1-\sqrt{1-\alpha }\right)$, the null hypothesis cannot be rejected, and it is reasonable to assume that the threshold value obtained from the regression is true [26].

## 3. Results

### 3.1. The Nonlinear Effect of Income on Food-Related Carbon Emissions

**Figure A2:**
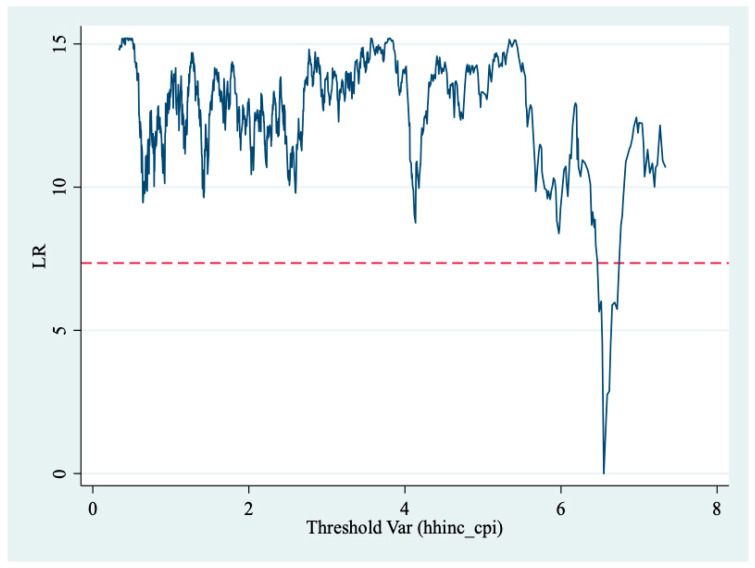
Threshold estimates and confidence intervals of household income. Note: The figure plots the LR statistic against alternative values of the household income threshold variable (hhinc). The solid line shows the LR statistic for each candidate threshold, and the dashed line marks the 95% critical value used to determine the confidence interval of the estimated threshold.

To study the threshold effect of household income on food-related carbon emissions, we first determine the number of thresholds that best characterize the model. We used 200 bootstrap replications for the threshold number test. The results reported in Table A1 show that only the single-threshold specification is statistically significant (*p* = 0.035), whereas the double- and triple-threshold specifications are not significant. We, therefore, proceed with the single-threshold model. Table A2 reports the estimated threshold value, and Figure A2 shows its 95% confidence interval.

The estimation results of Equation 1 are shown in Table 1. The estimated threshold reported in Table A2 is 6.5479, with a 95% confidence interval of [6.4965, 6.5917]. Because hhinc_cpi is measured in CPI-adjusted 10,000-yuan units, this threshold corresponds to an annual household income of 65,479 yuan. The household samples with incomes less than or equal to the threshold are classified into the low-income group, and the household samples with incomes higher than the threshold are classified into the high-income group. Household income and food-related carbon emissions are significantly and positively correlated in both groups, but the marginal effect of income on household food emissions is weaker than the threshold. For every 10,000-yuan increase in income, food emissions of low-income households increase by 0.0547 tons, whereas food emissions of high-income households increase by 0.0203 tons. Among the control variables, household size is significantly positively related to food-related emissions from food consumption, and the coefficients on age06, age65, and age are negative. The adjusted R^2^ of 0.243 indicates that substantial household-level variation remains unexplained, which is consistent with the role of preferences, habits, and other unobserved characteristics in shaping food-related emissions.

Although the regression coefficient of the right side of the threshold is smaller than the left, and the marginal effect of income on household food emissions decreases after exceeding the threshold, it is not enough to show that there is an EKC in food emissions. The theory of EKC suggests that environmental quality will tend to improve after the residents’ income reaches a certain level, and carbon emissions will decrease with the increase in income. However, there is currently insufficient evidence to suggest that there is an “*inflection point*” in Chinese residents’ food emissions, and further research is needed on the factors influencing food emissions on both sides of the threshold.

### 3.2. Income Impact on Food Consumption Amount

Food-related carbon emissions are directly related to food consumption. Table 2 reports annual per capita food consumption amounts and percentages for the high-income and low-income groups. High-income households consume more food overall (428.2 kg versus 400.1 kg), substantially less cereal and vegetables, and markedly more fruit and animal-source foods than low-income households. Animal food consumption in the high-income group is about 1.4 times that of the low-income group, and poultry consumption is nearly twice as high. Formal group-difference tests confirm that most of these amounts and share differences are statistically significant. 

Taking household food consumption amount as the dependent variable, we regress it on household income and the other control variables, and the results are shown in Table 3. For every 10,000-yuan increase in household income, the total food consumption amount of the full sample increases by about 4.5 kg on average. The increase is concentrated in low-income households, whose total food consumption rises by 16.4 kg, whereas the income effect for high-income households is statistically insignificant. This stable total consumption among high-income households helps explain why the post-threshold income effect on food-related emissions remains positive but becomes much smaller.

**Figure 2:**
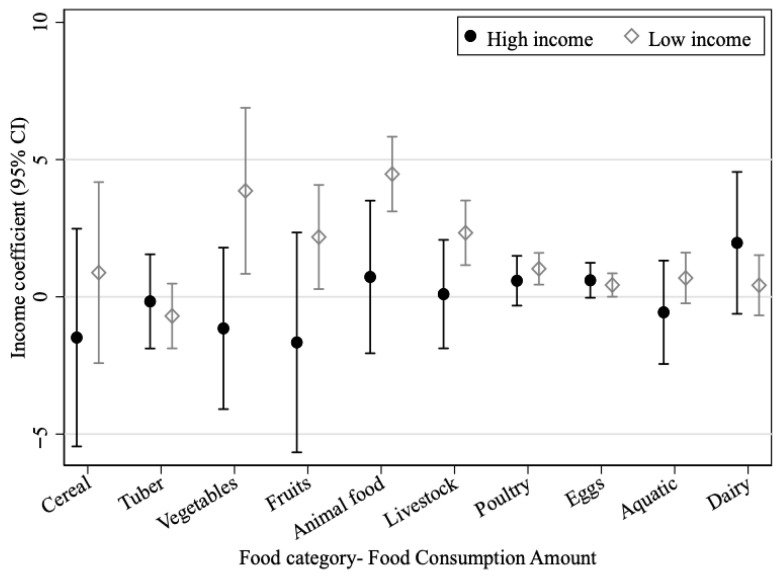
Income impact on the amount of food consumption.

Carbon emissions per unit differ widely across food categories, with meat products emitting substantially more than most plant-based foods. According to Poore and Nemecek [24], CO_2_ emissions are 51.7 kg CO_2_eq/kg for beef, 9.8 kg CO_2_eq/kg for pork, 7.8 kg CO_2_eq/kg for poultry, 4.2 kg CO_2_eq/kg for eggs, 2.3 kg CO_2_eq/kg for milk, 1.3 kg CO_2_eq/kg for wheat, and 3.1 kg CO_2_eq/kg for rice, while most vegetables and fruit are below 1 kg CO_2_eq/kg. Regressing category-specific food consumption amounts on household income, the results shown in Figure 2 indicate that income growth among low-income households significantly increases vegetable, fruit, and animal food consumption. For every 10,000-yuan increase in income, animal food consumption rises by about 4.5 kg, including roughly 2.3 kg of livestock meat and 1.0 kg of poultry.

It is also worth noting that income growth significantly increases the consumption of vegetables and fruit among low-income households, while the income effect on cereal consumption is no longer statistically significant. This pattern suggests that rising income among lower-income households is associated with a broader diet and a higher demand for relatively carbon-intensive foods.

### 3.3. Income Impact on Food Consumption Structure

**Figure 3:**
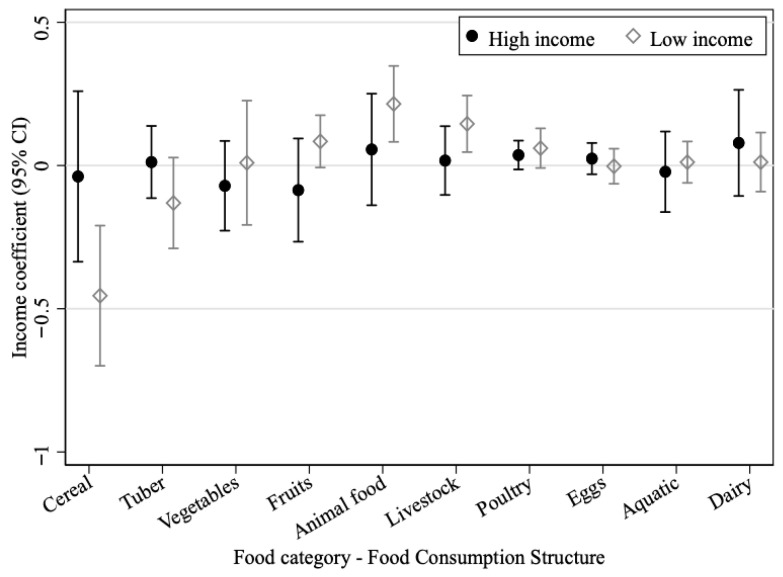
Income impact on food consumption structure.

In addition to changes in the amount of food consumption, changes in the structure of food consumption are also of equal interest. We regress the percentage share of each food category on household income, and the results are displayed in Figure 3.

For low-income households, the direction of the income effect on food-consumption shares is broadly consistent with the effect on absolute amounts. For every 10,000-yuan increase in income, the share of animal food rises by about 0.22 percentage points, the share of livestock rises by about 0.15 percentage points, and the share of cereal falls by about 0.45 percentage points. For high-income households, the income effects on food-consumption structure are statistically insignificant, which suggests a comparatively more stable dietary pattern within the sample period.

These results indicate partial convergence in dietary structure between income groups during the sample period rather than a complete convergence process. Because animal food generally has higher emissions per unit than plant food, the faster rise in animal-food demand among low-income households is consistent with a narrowing emissions gap across income groups. However, this sample-period pattern should not be interpreted as direct evidence that aggregate household food emissions will stabilize or decline in future periods.

### 3.4. Income Impact on Dietary Quality

After studying the income effect on household food consumption, the natural question is, does household dietary quality improve along with income growth? Clarifying the marginal effect of income on dietary quality can help to better predict the future trend of food-related carbon emissions. In addition, for policymakers, intervening in food overconsumption will have the dual effect of reducing carbon emissions and promoting the health of the population, while intervening in food under-consumption may be detrimental to the improvement of the quality of the population’s diets.

According to the recommended dietary intake provided by the Chinese Food Guide Pagoda (2022) [28], households with per capita daily food consumption below the lower limit of the recommended range are labeled as “insufficient”, and those above the upper limit are labeled as “excess”. The resulting household dietary quality is shown in Table 4. There is a serious imbalance in the food consumption of both groups: cereal is the most easily over-consumed food, with about 66.8% of high-income households and 74.3% of low-income households consuming cereal in excess; tuber crops, fruit, and dairy products are under-consumed by large majorities of households; and more than half of low-income households under-consume animal food. Under-consumption of fruit, animal food, and dairy products is less severe in the high-income group, whereas under-consumption of tuber crops and vegetables is more prevalent.

**Figure 4:**
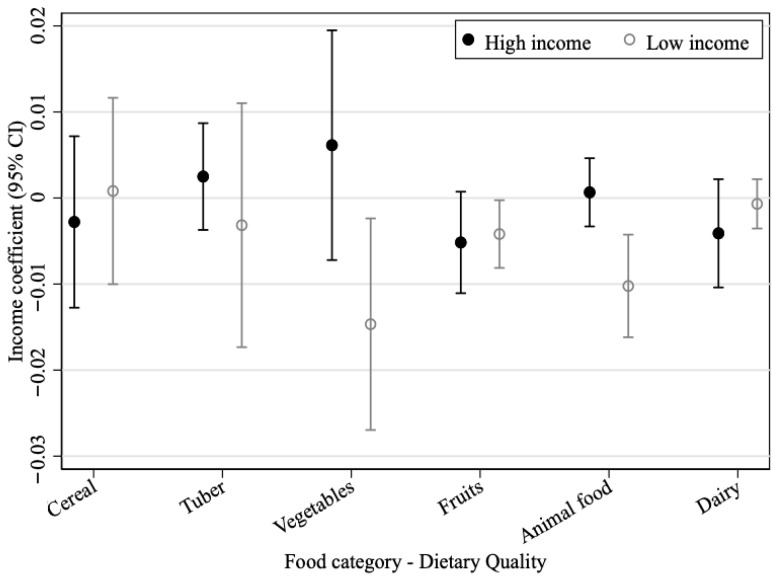
Income impact on food consumption quality.

Following He et al. [4], we calculate dietary deviation as zero when household consumption falls within the recommended interval; when consumption exceeds the upper bound, the difference between actual consumption and the upper bound is divided by the interval midpoint; and when consumption falls below the lower bound, the difference between the lower bound and actual consumption is divided by the same midpoint. Dietary deviation is therefore non-negative, with values closer to zero indicating better household dietary quality. Using these dietary deviations as the dependent variables, we regress them on household income and the other control variables, and the results are shown in Figure 4.

The marginal change in dietary quality is not the same for the two groups. For high-income households, income has no statistically significant effect on the quality of the six food categories considered here. For low-income households, higher income significantly worsens the dietary quality of vegetables, fruit, and animal food. The deterioration of vegetables and fruit mainly reflects persistent under-consumption, whereas the deterioration of animal food is more consistent with excessive intake.

In general, China’s food consumption, except for cereal, is under-consumed to a large extent, and the implementation of intervention policies such as carbon taxes on staple foods at this stage may affect the quality of residents’ diets, especially in low-income households, which would harm social equity. Information intervention policies to popularize the knowledge of “green and low-carbon” diets and green agricultural policies are more worthy of consideration under the current development conditions. Finally, it is worth noting that dairy products, as a food with high nutritional quality and relatively mild carbon emissions, are generally under-consumed by both groups of households, and the trend of improvement with income is not significant, which may be constrained by the dietary habits of the Chinese population. Policymakers can guide residents to increase their consumption of dairy products as a substitute for meat, which has higher carbon emissions per unit, and that will have the dual effect of improving dietary quality and reducing carbon emissions.

## 4. Discussion and Limitations

### 4.1. Discussion

The 2004–2011 sample should be interpreted as a historically specific transition window rather than as a direct description of current Chinese households. Public data show that this was the period when rapid income growth, declining Engel coefficients, and the expansion of animal-source food consumption occurred simultaneously. For that reason, the sample is especially informative for identifying how rising income reshaped household food demand, dietary quality, and food-related carbon emissions during the active phase of dietary upgrading.

The threshold result is better understood as nonlinear heterogeneity than as evidence of an EKC turning point. Below the threshold, additional income is associated with faster growth in total food consumption and animal-source food demand; above the threshold, the marginal effect remains positive but becomes smaller. This pattern is consistent with partial demand saturation, but it may also reflect differences in preferences, habits, and nutritional upgrading rather than any automatic decarbonization mechanism.

The dietary-quality results reinforce this interpretation. Higher income among low-income households is associated with more animal food and fruit consumption, but it does not automatically produce a lower-carbon or fully balanced diet. The findings therefore suggest that the same process of dietary upgrading can improve some dimensions of diet while simultaneously intensifying carbon pressure. For that reason, the evidence should be treated as a mechanism-based interpretation of the 2004–2011 transition rather than as direct proof that future household food emissions will stabilize on their own.

### 4.2. Limitations

This study has several limitations. First, the sample period ends in 2011, so the results should be interpreted as evidence for a historically specific transition stage rather than as a direct description of present-day Chinese households. Although CHNS remains the most authoritative large-scale micro food-consumption dataset available for China, the 2015 CHNS does not provide the food-consumption module needed to extend the present analysis within the same household-level framework.

Second, household food-related carbon emissions are constructed from indirect global median emission factors rather than from direct observation of all household food-system activities. The analysis is therefore best suited to identifying the structure of dietary transition rather than to measuring every channel of food-related emissions, and the benchmark factors may not fully capture China-specific production conditions, technologies, and supply chains.

Third, the CHNS food-consumption module is based on a 72 h dietary recall. Although this method is standard in nutrition surveys, short recall windows may not fully capture each household’s longer-run dietary patterns, which introduces measurement error into the food-intake variables and the derived carbon-emission indicators.

Fourth, CHNS covers 12 provinces across eastern, central, and western China, but it is not nationally representative of all regions. The geographic scope of the sample should therefore be kept in mind when interpreting the findings, especially given the substantial changes in China’s food environment since 2011, including online food delivery, away-from-home eating, processed foods, cold-chain logistics, and broader supply-chain upgrading.

## 5. Conclusions and Policy Implications

### 5.1. Conclusions

Using CHNS household panel data for 2004–2011, this study identifies a nonlinear threshold relationship between income and household food-related carbon emissions in China. The main contribution of the analysis is not that it describes the current level of Chinese food emissions, but that it captures the active phase of dietary upgrading in which rising income, changing food structure, and increasing carbon pressure became tightly linked at the household level.

We find no evidence of a short-term EKC turning point in China’s food-related carbon emissions. Household income remains positively associated with food-related emissions, although the marginal effect declines once annual household income reaches 65,479 yuan. Among low-income households, income growth is associated with higher total food consumption and faster growth in relatively carbon-intensive foods, whereas the income effects on food-consumption amount and structure are statistically insignificant for high-income households during the sample period. These results indicate heterogeneity in dietary responses across income groups, but they should not be read as direct evidence that aggregate household food emissions will stabilize automatically over time.

### 5.2. Policy Implications

The policy implications of this study should be interpreted in light of the sample period. The results identify a structural relationship between income growth, dietary upgrading, and food-related carbon emissions during a critical transition stage in China; they should not be read as a mechanical forecast for present-day households. Even so, the evidence shows that food-related emissions do not decline automatically as incomes rise and that household dietary upgrading can intensify carbon pressure when it relies heavily on animal-source foods.

Policy responses should not be framed as shifting carbon-intensive food production abroad. Instead, the priority is to reduce emissions within China’s own food system while safeguarding food security. This requires continued support for low-carbon agricultural technologies, cleaner energy use in food production and logistics, more resilient domestic supply chains, and dietary guidance that encourages nutritionally balanced and lower-carbon food choices.

China’s food policy should also be continuously adjusted to guide the green transformation of the population’s food consumption. In the context of improving the lives of the population, “green and low-carbon” should be incorporated into national dietary guidelines and publicized. Foods such as livestock meat have significantly higher carbon emissions than plant foods and dairy products, which provide the same amount of protein. Residents should be encouraged to reduce their meat diets and increase their consumption of dairy products and plant foods, leading to a shift in their food consumption structure towards “less meat, more milk”.

Finally, against the backdrop of relatively stable food demand, carbon emissions reduction on the food production side needs to be given sufficient attention. China should actively promote carbon emissions reduction in the agricultural sector, improve the efficiency of energy utilization in the agricultural sector, and increase guidance and support for green energy technologies and energy-saving technologies at the policy level, including financial subsidies, tax exemptions, and other means, to form a benign development of the industry.

## Figures and Tables

**Table 1:** Estimation results of the threshold effect.

Variables	Coefficient	t Statistic
Income (low-income group)	0.0547 **	(2.58)
Income (high-income group)	0.0203 *	(2.16)
hhsize	0.935 ***	(10.09)
age06	−0.153 *	(−1.93)
age65	−0.0507	(−1.49)
Age	−0.00731	(−1.77)
edu	0.0110	(1.14)
Marriage	−0.143 *	(−1.87)
_cons	0.715	(1.50)
N	8288	
adj. R^2^	0.243	

T-statistics in parentheses. * *p* < 0.10, ** *p* < 0.05, *** *p* < 0.01.

**Table 2:** Food consumption per capita in both groups.

Food Category	High Income		Low Income		Group Difference	
	Amount (kg)	Percentage (%)	Amount (kg)	Percentage (%)	Amount *p*-Value	Percentage *p*-Value
Overall	428.2	100	400.1	100	0.003	.
Cereal	135.4	33.3	149.2	38.9	0.002	0.000
Tuber crop	12.7	3.0	15.1	3.7	0.168	0.107
Vegetables	112.7	26.4	120.7	30.0	0.036	0.000
Fruit	30.1	6.1	18.0	3.8	0.000	0.000
Animal food *	68.2	15.9	49.7	12.3	0.000	0.001
(Livestock)	32.0	7.5	24.4	6.1	0.000	0.002
(Poultry)	8.6	2.0	4.4	1.0	0.002	0.003
(Eggs)	12.5	3.0	10.3	2.6	0.007	0.034
(Aquatic products)	15.1	3.4	10.7	2.5	0.007	0.020
Dairy products	11.2	2.4	4.6	1.1	0.001	0.001

* Animal food includes livestock, poultry, eggs, and aquatic products. Group-difference tests are reported using cluster-robust standard errors at the provincial level. The percentage *p*-value is not reported overall because the percentage is mechanically equal to 100 for both groups.

**Table 3:** Income impact on the total food consumption amount.

Variable	(1)	(2)	(3)
	All	High Income	Low Income
hhinc	4.519 ***	0.0207	16.38 ***
	(3.62)	(0.00)	(8.12)
Control variable	Yes	Yes	Yes
N	8288	1056	7232
adj. R^2^	0.423	0.304	0.434

T-statistics in parentheses. *** *p* < 0.01

**Table 4:** Quality of household diets.

Category	Recommended Consumption (g/Day)	High Income			Low Income		
		Average Consumption (g/Day)	Insufficient (%)	Excess (%)	Average Consumption (g/Day)	Insufficient (%)	Excess (%)
Cereal	200–300	371.1	6.7	66.8	408.7	4.2	74.3
Tuber	50–100	34.9	74.0	9.3	41.4	68.9	13.5
Vegetables	300–500	308.8	55.1	11.0	330.8	49.3	14.5
Fruit	200–350	82.4	85.6	3.4	49.2	92.0	2.1
Animal food	120–200	186.9	29.9	39.7	136.3	51.0	23.0
Dairy	300–500	30.7	99.5	0	12.6	99.7	0.0

## Data Availability

This research uses data from the China Health and Nutrition Survey (CHNS), which is available from http://www.cpc.unc.edu/projects/china (accessed on 25 June 2025). We thank the National Institute of Nutrition and Food Safety, China Center for Disease Control and Prevention, Carolina Population Center, the University of North Carolina at Chapel Hill, the NIH (R01-HD30880, DK056350, and R01-HD38700) and the Fogarty International Center, NIH for financial support for the CHNS data collection and analysis files from 1989 to 2006 and both parties plus the China-Japan Friendship Hospital, Ministry of Health for support for CHNS 2009 and future surveys.

## Appendix A

**Table A1:** Threshold existence test.

Threshold Tests	F-Value	*p*-Value	Critical Values		
			1%	5%	10%
Single threshold	15.2208	0.0350	18.4984	13.0706	11.6601
Double threshold	6.0421	0.6050	17.3823	13.9269	12.0731
Triple threshold	10.3496	0.3850	30.3135	23.9461	17.9059

Source(s): Authors’ work

**Table A2:** Household income threshold estimate.

Threshold	Estimate	95% Confidence Interval
Income threshold	6.5479	[6.4965, 6.5917]

Source(s): Authors’ work
